# Fabrication of porous NiMn_2_O_4_ nanosheet arrays on nickel foam as an advanced sensor material for non-enzymatic glucose detection

**DOI:** 10.1038/s41598-019-54746-2

**Published:** 2019-12-02

**Authors:** Jie Zhang, Yudong Sun, Xianchun Li, Jiasheng Xu

**Affiliations:** 1 School of Chemical Engineering, University of Science and Technology Liaoning, Anshan, 114051 P.R. China; 2 Liaoning Province Key Laboratory for Synthesis and Application of Functional Compounds, College of Chemistry and Chemical Engineering, Bohai University, Jinzhou, 121013 P.R. China

**Keywords:** Sensors and biosensors, Sensors

## Abstract

In this work, porous NiMn_2_O_4_ nanosheet arrays on nickel foam (NiMn_2_O_4_ NSs@NF) was successfully fabricated by a simple hydrothermal step followed by a heat treatment. Porous NiMn_2_O_4_ NSs@NF is directly used as a sensor electrode for electrochemical detecting glucose. The NiMn_2_O_4_ nanosheet arrays are uniformly grown and packed on nickel foam to forming sensor electrode. The porous NiMn_2_O_4_ NSs@NF electrode not only provides the abundant accessible active sites and the effective ion-transport pathways, but also offers the efficient electron transport pathways for the electrochemical catalytic reaction by the high conductive nickel foam. This synergy effect endows porous NiMn_2_O_4_ NSs@NF with excellent electrochemical behaviors for glucose detection. The electrochemical measurements are used to investigate the performances of glucose detection. Porous NiMn_2_O_4_ NSs@NF for detecting glucose exhibits the high sensitivity of 12.2 mA mM^−1^ cm^−2^ at the window concentrations of 0.99–67.30 μM (correlation coefficient = 0.9982) and 12.3 mA mM^−1^ cm^−2^ at the window concentrations of 0.115–0.661 mM (correlation coefficient = 0.9908). In addition, porous NiMn_2_O_4_ NSs@NF also exhibits a fast response of 2 s and a low LOD of 0.24 µM. The combination of porous NiMn_2_O_4_ nanosheet arrays and nickel foam is a meaningful strategy to fabricate high performance non-enzymatic glucose sensor. These excellent properties reveal its potential application in the clinical detection of glucose.

## Introduction

The World Health Organization (WHO) reported that the diabetes and its complications by high glucose concentration in the human body have resulted in approximately 350 million people around world^1–3^. The diabetes will be the 7^th^, leading cause of death by 2030^3–5^. Development of highly selective and sensitive detection method for glucose detection is of significance in a variety of fields, such as blood glucose testing, foods monitoring and pharmaceutical analysis^5–7^. The conventional techniques, such as colorimetry, chemiluminescence, electro-chemiluminescence and fluorescence are used as determination method for glucose biomolecule^7–10^. With the rapid developments of the electrochemical science and technology, electrochemical sensors have been developed to detect glucose levels due to its combination of performance parameters such as reliable, accurate, sensitive and fast response^10–14^.

Sensors for glucose detection, including enzymatic and non-enzymatic detection methods, are the most convenient and promising method due to its sensitivity and reproducibility^15,16^. Enzyme-immobilized glucose sensors suffer from some intrinsic drawbacks such as lack of temperature and pH stability, high cost and low shelf-life^17–19^. The above mentioned drawbacks limit their practical application although they possess high sensitivity and the excellent selectivity^20,21^. Non-enzymatic glucose sensors can avoid the intrinsic drawbacks of enzyme sensor. Non-enzymatic electrochemical glucose sensors possess the advantages over enzymatic sensors in terms of low cost, good thermal stability and satisfactory reproducibility^21,22^. Non-enzymatic electrochemical detection sensor is based on the oxidize glucose to a detectable electrochemical signal, which directly occurs on the electrode surface by an electric current effect^23^. For the non-enzymatic glucose sensor, the design and fabrication of the active materials play a key role in the sensing performances since it can efficiently catalyze and oxidize glucose to produce gluconolactone and can free from the operating conditions^24,25^.

In recent years, many materials including carbon materials, noble metals, transition metals and transition metal oxides have been widely investigated and used as the active materials in non-enzymatic glucose sensor^26–28^. The poor biocompatibility and low electrical conductivity of carbon materials and high price of noble metals hinder their practical applications^29–31^. Among them, transition metal oxide materials have been extensively explored and researched as electrode materials due to their excellent properties, such as the featured shape dependent, good biocompatibility, non-toxic, electro-catalytic properties and low cost compared with other materials^31–34^. NiMn_2_O_4_, a transition metal oxide with the cubic spinel structure, has attracted attention owing to its high electrical conductivity, good electrochemical performances, abundance in nature, low cost, low toxicity and environmental friendliness. NiMn_2_O_4_ shows a potential as electrode material due to its strong electro-site based on generation of two redox couples of Ni^2+^/Ni^3+^ and Mn^3+^/Mn^4+^ in alkaline solution^35,36^. NiMn_2_O_4_ as a promising material will exhibit an excellent electrochemical activity toward the electrochemical detection of glucose.

The design and use of nanomaterials have increasing interest in the fields of glucose detection and sensing^37^. Use of nanostructured materials effectively improves the material characteristics, such as, electron/ion transfer rate, adsorption capability and high loading and immobilization of biomolecules. The nanostructured materials as the ideal candidates overcome drawbacks and limitations and lead to the development of glucose sensor with fast response, high sensitivity and selectivity. For sensors, the active materials intensively depend on the amperometric response of glucose oxidation at the surface of active materials. The nanostructure of the active materials can enlarge the surface-to-volume ratio and enhance the accessible electrochemical reactive sites. The nanostructures also efficiently shorten the transport distance of ions and charges between electrolyte and surface of active materials^38–40^. Thus, developing a nanostructured NiMn_2_O_4_ electrode as non-enzymatic material is of significance to improve the sensing performances of direct glucose detection.

In this work, porous NiMn_2_O_4_ NSs@NF sensor electrode has been designed and fabricated by a facile hydrothermal method followed by a heat treatment. We have chosen 3D nickel foam as electrode substrate for the direct growth of porous NiMn_2_O_4_ nanosheet arrays since nickel foam possesses the advantages of commercial availability, low cost, the excellent electrical conductivity and porous structure, which provides large surface area for the nanostructured construction. This direct grown structure of porous NiMn_2_O_4_ NSs@NF provides unique properties, such as good electron transport, reduced resistance, the excellent electrical conductivity and better adhesion stability compared with non-direct grown electrode. In addition, a synergistic effect between porous NiMn_2_O_4_ nanosheets and the metal substrate enables NiMn_2_O_4_ NSs@NF electrode with the excellent sensing performances for glucose molecules. Porous NiMn_2_O_4_ NSs@NF sensor exhibits a high sensitivity of 12.2 mA mM^−1^ cm^−2^ at the window concentrations of 0.99–67.30 μM and 12.3 mA mM^−1^ cm^−2^ at the window concentrations of 0.115–0.661 mM. Porous NiMn_2_O_4_ NSs@NF electrode also exhibits a low LOD of 0.24 µM as well as the fast response of 2 s. These results suggest that porous NiMn_2_O_4_ NSs@NF is an efficient sensor electrode for glucose detection.

## Result and Discussions

**Figure 1:**
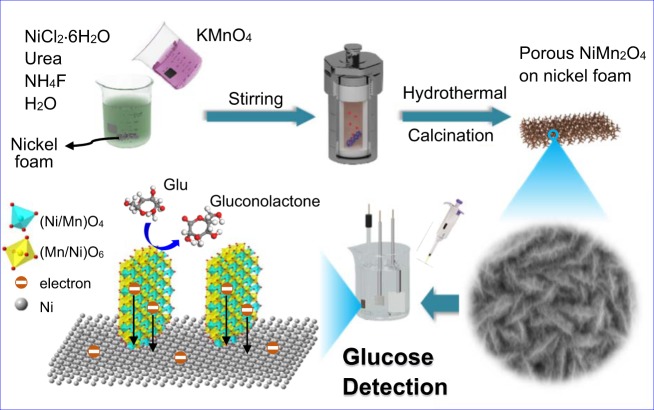
Schematic illustrations of the fabrication process for porous NiMn_2_O_4_ NSs@NF sensor electrode.

Figure 1 illustrates the scheme for the representative fabrication process of porous NiMn_2_O_4_ NSs@NF sensor electrode. The fabrication process of porous NiMn_2_O_4_ NSs@NF electrode includes a hydrothermal process and a heat treatment process. The fabricated porous NiMn_2_O_4_ NSs@NF is directly used as a sensor electrode in the glucose detection system.

**Figure 2:**
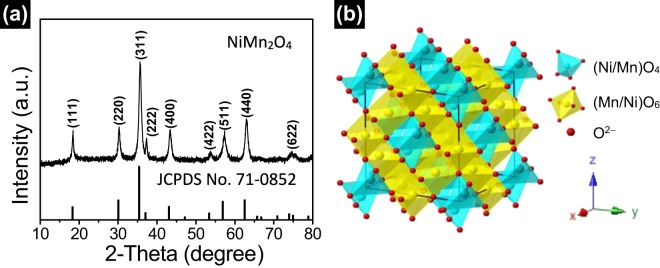
(**a**) XRD patterns of porous NiMn_2_O_4_ NSs@NF sensor electrode. Several vertical lines at the bottom of XRD patterns is the standard XRD diffraction peaks from JCPDS card No. 71-0852. (**b**) Schematic cystal structure of the spinel NiMn_2_O_4_. Skyblue color tetrahedra denotes the (Ni/Mn)O_4_ tetrahedra, yellow color octahedra denotes the (Mn/Ni)O_6_ octahedra.

XRD patterns are performed to identify the crystallographic structure and the chemical compositions of porous NiMn_2_O_4_ nanosheet arrays (NiMn_2_O_4_ nanosheets are scraped from the nickel foam electrode). XRD patterns of porous NiMn_2_O_4_ NSs@NF are shown in Fig. 2. The diffraction peaks at 2*θ* values of 18.3°, 30.1°, 35.4°, 37.0°, 43.0°, 53.2°, 56.9°, 62.5°, 73.9° and 74.9° are observed, which can be well indexed to the (111), (220), (311), (222), (400), (422), (511), (440), (533) and (622) planes of the spinel-type NiMn_2_O_4_ phase, respectively. These diffraction peaks can be well indexed to the peaks of the NiMn_2_O_4_ standard diffraction patterns (JCPDS PDF No. 71-0852, *a* = *b* = *c* = 8.4 Å, space group: cubic *Fd-3m*(227), Z = 8)^41^, indicating the high purity of NiMn_2_O_4_ phase. These diffraction peaks are broaden in width and weaken in intensity, indicating the low crystallinity of the NiMn_2_O_4_ sample^42^. Figure 2b shows the crystal structure of the spinel NiMn_2_O_4_ sample. Nickel ions and manganese are adopted the cubic structure with mixed valence states for the spinel NiMn_2_O_4_ structure; nickel and manganese occupy randomly but totally proportionally (Ni:Mn = 1:2) in the interstices of oxygen stacking tetrahedron and octahedron^43,44^. These cations occupy the cubic lattice composing by close-packed oxygen anions (O^2−^)^45^. For the spinel NiMn_2_O_4_ materials, the low crystalline can improve their electrochemical performances due to numerous of loosely packed atoms being available for redox reaction^46^.

**Figure 3:**
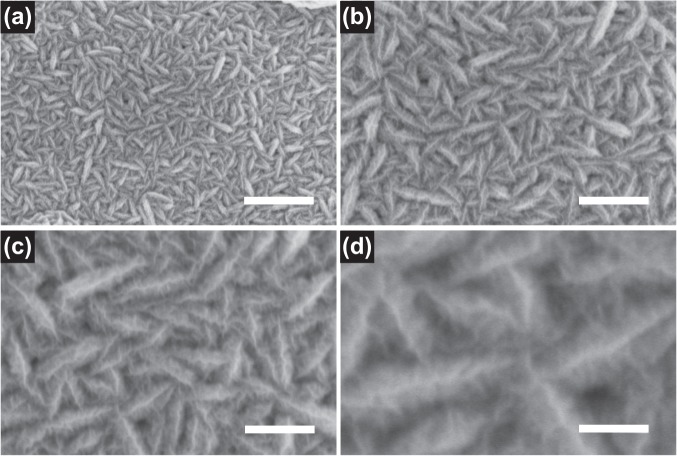
SEM images of porous NiMn_2_O_4_ NSs@NF sensor electrode. (**a**) Low magnification SEM image of porous NiMn_2_O_4_ NSs@NF, scale bars = 1 μm. (**b,c**) Medium magnification SEM images of porous NiMn_2_O_4_ NSs@NF, scale bars = 500 μm and 300 nm, respectively. (**d**) High magnification SEM image of porous NiMn_2_O_4_ NSs@NF, scale bars = 100 nm.

The morphology and structure of porous NiMn_2_O_4_ NSs@NF sensor electrode are characterized by SEM. Different magnification SEM images of porous NiMn_2_O_4_ NSs@NF are shown in Fig. 3. Low magnification SEM image shows a panoramic view (Fig. 3a). Porous NiMn_2_O_4_ nanosheets are completely and uniformly coated on surface of nickel foam substrate. Porous NiMn_2_O_4_ nanosheets and nickel foam substrate form an open arrays structure composed of numerous NiMn_2_O_4_ nanoparticles with uniform size. Figure 3b,c show medium magnification SEM images of porous NiMn_2_O_4_ NSs@NF. From Fig. 3b, the porous NiMn_2_O_4_ nanosheets are cross-linked each other and vertically anchored on the surface of nickel foam substrate to form three dimensional ordered NiMn_2_O_4_ nanosheet arrays. From the Fig. 3c, the length of porous NiMn_2_O_4_ nanosheets is about 250 nm and the breadth of porous NiMn_2_O_4_ nanosheets is about 50 nm. Moreover, the skeleton surface of NiMn_2_O_4_ nanosheets is completely coated by the smaller NiMn_2_O_4_ nanosheets. This hierarchical NiMn_2_O_4_@NiMn_2_O_4_ core-shell nanosheet nanostructure provides larger surface area, which is capable of facilitating the electrolyte ions diffusion and increasing the contact area between the electrode materials and the electrolyte ions.

Figure 3d shows the high magnification SEM image of porous NiMn_2_O_4_ NSs@NF. These smaller NiMn_2_O_4_ nanosheets growth on surface of NiMn_2_O_4_ nanosheets. In addition, we also can find that these smaller NiMn_2_O_4_ nanosheets are almost vertical to the surface of NiMn_2_O_4_ nanosheet. These smaller NiMn_2_O_4_ nanosheets are wrinkles. The length of these smaller NiMn_2_O_4_ nanosheets is about 20 nm on average. More detailed structures are further shown in Figs. S1–S4. There is space between the smaller NiMn_2_O_4_ nanosheets forming 3D spatial structure, which can facilitate the electrolyte ions diffusion and provide larger surface area for electrocatalytic reactions. The unique 3D core-shell structure greatly reduces distance for the diffusion/transport of electrolyte ions, which can be attributed to the opening structure and excellent performances of NiMn_2_O_4_ nanosheets.

**Figure 4:**
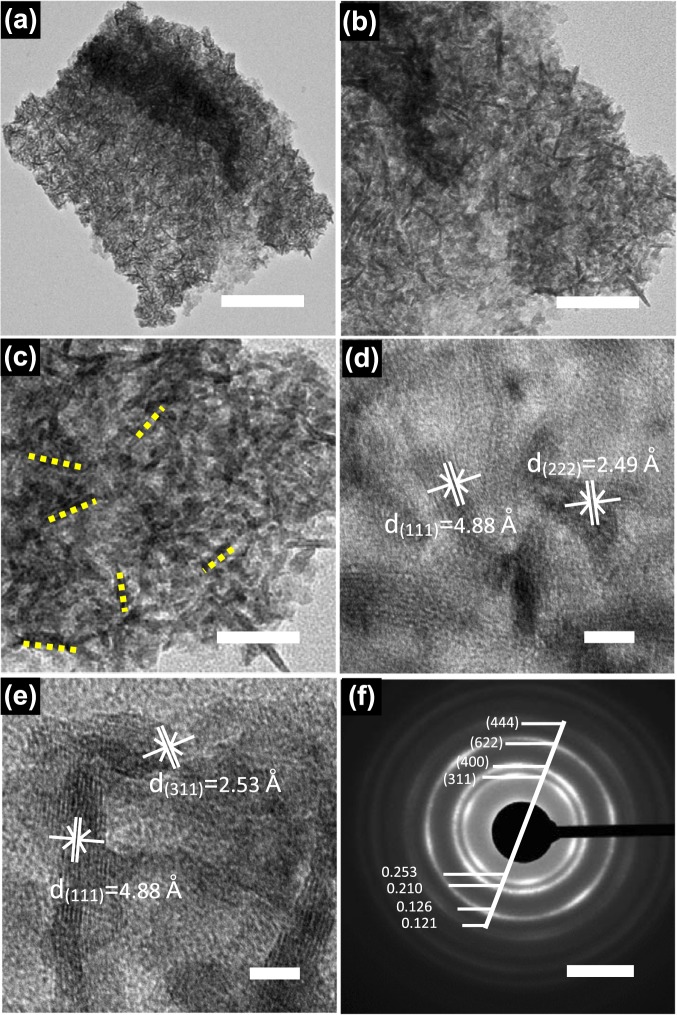
TEM images of porous NiMn_2_O_4_ NSs@NF sensor electrode. (**a**) Low magnification TEM image, scale bar = 200 nm; (**b**) Medium magnification TEM image, scale bar = 100 nm; (**c**) High magnification TEM image, scale bar = 50 nm; (**d,e**) High resolution TEM (HRTEM) image of porous NiMn_2_O_4_ NSs@NF, scale bar = 5 nm; (**f**) The selected area electron diffraction (SAED) patterns of porous NiMn_2_O_4_ NSs@NF, scale bar = 5 1/nm.

Figure 4 shows TEM and HRTEM images of porous NiMn_2_O_4_ nanosheet arrays (porous NiMn_2_O_4_ nanosheets are scratched from nickel foam consisted of stacking nanosheets). Figure 4a displays a low magnification TEM image. An overall contour of NiMn_2_O_4_ nanosheet is observed in the low magnification TEM image. From this low magnification TEM image, the length and width of this porous NiMn_2_O_4_ nanosheet can be clearly seen about 600 nm and 400 nm, respectively. Figure 4b shows medium magnification TEM image. Medium magnification TEM image further shows the unique core-shell structure of the NiMn_2_O_4_ nanosheet arrays. Figure 4c shows high magnification TEM image. This unique core-shell structure can be clearly observed in high magnification TEM image. These smaller NiMn_2_O_4_ nanosheets, called as “shell” nanosheets, are distributed uniformly on the surface of the “core” NiMn_2_O_4_ nanosheet. Some “shell” NiMn_2_O_4_ nanosheets are marked by the yellow dash line in Fig. 4c. The thickness of the “shell” NiMn_2_O_4_ nanosheets is about 10 nm. This core-shell structure can effectively provide lager surface area and markedly shorten the ion diffusion distance^47^. Figures S5–S7 shows more detailed structures.

Figure 4d,e show the typical high resolution TEM (HRTEM) images of porous NiMn_2_O_4_ nanosheet. Two lattice fringes with the interplanar *d*-spacing of the 4.88 and 2.49 Å (marked by white) are observed in Fig. 4d, which can be well indexed to the (111) and (222) planes of NiMn_2_O_4_, respectively. Figure 4e also shows two lattice fringes with the *d*-spacing of the 4.88 and 2.53 Å, which are well indexed to the (111) and (311) planes of NiMn_2_O_4_, respectively. These lattice fringes are agrees well with the XRD patterns (Fig. 2). The selected area electron diffraction (SAED) patterns of porous NiMn_2_O_4_ nanosheet are shown in Fig. 4f. The SAED patterns are composed of several light diffraction circles. Four major diffraction circles can be observed on the SAED patterns. These major diffraction circles clearly match with the (444), (622), (400) and (311) planes of porous NiMn_2_O_4_ nanosheet, respectively, representing the existence of porous NiMn_2_O_4_ nanosheet and its polycrystalline structure^48^.

**Figure 5:**
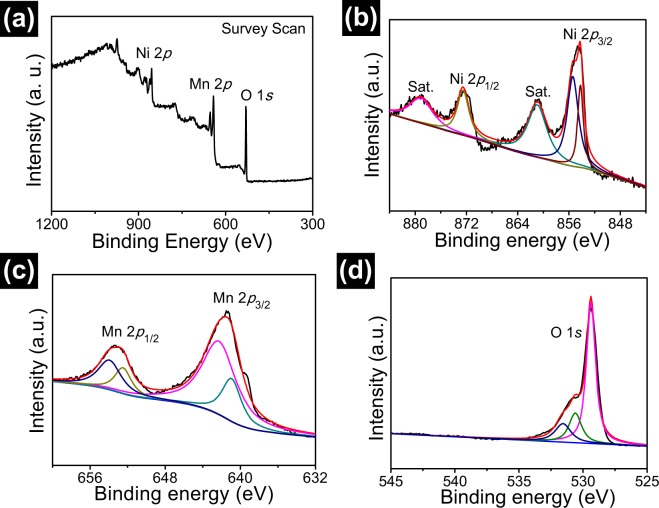
(**a**) X-ray photoelectron spectroscopy (XPS) survey scan spectra of porous NiMn_2_O_4_ NSs@NF sensor electrode. (**b**) Ni 2*p* spectrum. (**c**) Mn 2*p* spectrum. (**d**) O 1 *s* spectrum. These black and red curves correspond to the experimental and fitted curves of the spectra of Ni 2*p*, Mn 2*p* and O 1 *s*.

To further analyze the elemental composition and oxidation state, porous NiMn_2_O_4_ NSs@NF sensor electrode is characterized by XPS and the results are analyzed with based on Gaussian-Lorentzian fitting method. Figure 5a displays the full XPS survey spectra of porous NiMn_2_O_4_ NSs@NF electrode, which mainly contains the elements of Ni, Mn and O. Figure 5b shows the Ni spectrum of porous NiMn_2_O_4_ NSs@NF electrode. Two peaks with the binding energies at 853.9 and 855.4 eV correspond to the Ni 2*p* 3/2^49,50^. The peak at 872.6 eV corresponds to the Ni 2*p* 1/2^51^. Two peaks with the binding energies located at 860.9 and 879.3 eV as shown in Fig. 5b are the satellite (Sat.) peaks of the Ni 2*p* 3/2 and Ni 2*p* 1/2, respectively^52^. Figure 5c shows Mn spectrum of porous NiMn_2_O_4_ NSs@NF electrode. Two spin-orbit peaks in Mn spectrum are deconvolved into four peaks. Two deconvolved peaks are observed at 641.0 and 642.5 eV, which correspond to Mn 2*p* 3/2. Two deconvolved peaks are observed at 654.0 and 652.6 eV, corresponding to Mn 2*p* 1/2, which is consistent with the previous reported literature^53^. Two deconvolved peaks are observed at 641.0 and 652.6 eV, which correspond to the correlative peaks of Mn^2+^; two deconvolved peaks are observed at 642.5 and 654.0 eV, which correspond to the correlative peaks of Mn^3+^ binding energy^54^. Figure 5d shows the O spectrum of porous NiMn_2_O_4_ NSs@NF electrode. The resolved peak at binding energy of 529.4 eV is indexed to typical metal oxygen bonds (M-O-M) or the lattice oxygen^55–57^. The peak for O 1 *s* at 530.6 eV is attributed to metal-O-H from metal surface hydroxyl groups^33,58^. The peak at 531.6 eV is attributed to a larger number of defect sites with a low oxygen coordination normally observed in materials with small particles^55^. The energy dispersive spectroscopy (EDS) mappings of porous NiMn_2_O_4_ NSs@NF sensor electrode are shown in Fig. S8. These EDS mappings indicate that Ni, Mn and O elements are uniformly distributed on porous NiMn_2_O_4_ nanosheet arrays, which in agreement with XRD and XPS characterizations.

The surface area and porosity are two important factors, which can critically influence the sensing performances for detection of glucose. The surface area and porosity of the porous NiMn_2_O_4_ NSs@NF sensor electrode is further analyzed by Brunauer–Emment–Teller (BET) nitrogen isothermal adsorption and desorption test. Figure S9 shows a typical BET nitrogen adsorption and desorption isotherms of NiMn_2_O_4_ NSs@NF electrode. Nitrogen adsorption and desorption isotherms are plotted as quantity volume (*V*_*m*_) on the y-axis and relative pressure (*P/P*_0_) on the x-axis based to BET experimental data. According to the BET test, the BET specific surface area of porous NiMn_2_O_4_ NSs@NF electrode is calculated to be 77.5 m^2^ g^−1^. This large specific area can effectively increase the utilization of NiMn_2_O_4_ as an electrochemically active material in the process of glucose electrochemical detection. The adsorption/desorption isotherms also show a hysteresis, which can be classified as a type IV isotherm according to the profile of the hysteresis loop in a range of 0.5 < *P/P*_0_ < 1.0^59^. Inset in Fig. S9 shows the corresponding pore-size distribution with calculated by the Barrette Joynere Halenda (BJH) method based on BET experimental data. The pore size distribution image shows a wide pore-size distribution characteristic, which can be attributed to the porous nano-structure of NiMn_2_O_4_ NSs@NF electrode. The pore size distribution image presents that the average pore size of NiMn_2_O_4_ NSs@NF electrode is about 9.6 nm. Porous structure of NiMn_2_O_4_ NSs@NF electrode with large surface area provides the highway for transportation of electrons and ions between the electrolytes and electrode material, which is in favour of non-enzymatic glucose detection.

**Figure 6:**
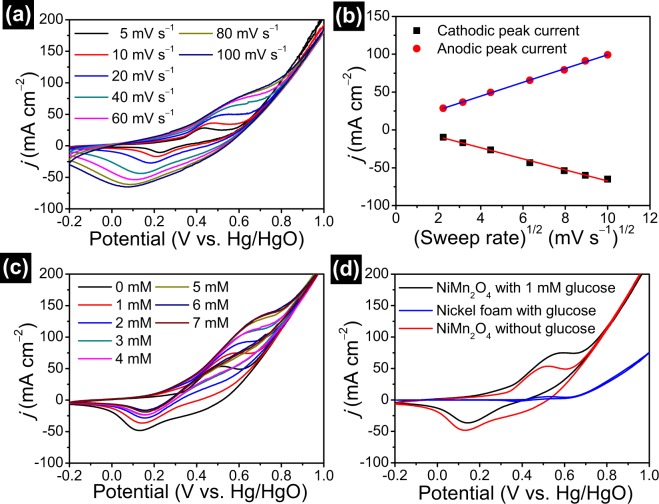
(**a**) CV curves of porous NiMn_2_O_4_ NSs@NF sensor electrode in 0.5 M NaOH electrolyte with 1.0 mM glucose at the sweep rate range from 10 to 100 mV s^−1^. (**b**) The corresponding fitting curves of anode and cathode peak response currents as a function of the square root of sweep rates. (**c**) CV curves of porous NiMn_2_O_4_ NSs@NF in 0.5 M NaOH electrolyte with concentrations of glucose range from 0 to 7 mM at a sweep rate of 20 mV s^−1^. (**d**) CV curves of porous NiMn_2_O_4_ NSs@NF and pure nickel foam at the same sweep rate of 20 mV s^−1^ in 0.5 M NaOH electrolyte with 1.0 mM glucose and without 1.0 mM glucose.

Porous NiMn_2_O_4_ NSs@NF electrode is used directly as a sensor electrode to test its electrocatalytic activity toward glucose detection in 0.5 M NaOH electrolyte. Figure 6a presents CV curves of porous NiMn_2_O_4_ NSs@NF electrode at sweep rate ranging from 10 to 100 mV s^−1^. Each CV curve displays a pair of redox peak. These peaks in CV curves can be attributed to the electrochemical redox reactions/electrocatalytic oxidation reactions of porous NiMn_2_O_4_ NSs@NF electrode. The electrocatalytic oxidation reaction for glucose detection are shown as follows formulae (1–4)^60–62^:

1 $${{\rm{NiMn}}}_{2}{{\rm{O}}}_{4}+{{\rm{OH}}}^{-}+{{\rm{H}}}_{2}{\rm{O}}\leftrightarrow {\rm{NiOOH}}+{\rm{2MnOOH}}+{{\rm{e}}}^{-}$$

2 $${{\rm{OH}}}^{-}+{\rm{MnOOH}}\leftrightarrow {{\rm{H}}}_{2}{\rm{O}}+{{\rm{MnO}}}_{2}+{{\rm{e}}}^{-}$$

3 $${{\rm{Ni}}}^{3+}+{\rm{glucose}}\to {{\rm{Ni}}}^{2+}+{\rm{gluconolactone}}$$

4 $${{\rm{Mn}}}^{4+}+{\rm{glucose}}\to {{\rm{Mn}}}^{3+}+{\rm{gluconolactone}}$$

As shown in CV curve at sweep rate of 10 mV s^−1^, a pairs of redox peaks at +0.23/+0.43 V can be observed. With the increase of scan rates, the positive potential shift of the anodic peaks and negative potential shift of cathodic peaks are also observed. The separation of peak to peak (ΔEp) increases linearly with the increasing scan rates. These phenomena may be attributed to the increase of overpotential^63^. Figure 6b shows the corresponding fitting curves of the response currents vs. the square root of sweep rates. The corresponding fitting curves show the linear dependencies on the sweep rates, indicating that the electron transfer process of electrode is the reversible and diffusion-controlled electrochemical redox process^64^.

CV curves of porous NiMn_2_O_4_ NSs@NF sensor electrode at various concentrations of glucose are recorded to investigate the electrochemical sensing performance at the sweep rate of 20 mV s^−1^ in a 0.5 M NaOH electrolyte solution. Figure 6c shows CV curves of porous NiMn_2_O_4_ NSs@NF electrode at various concentrations of glucose (from 0 mM to 7 mM). The response currents show a steadily increasing trend with the increase of glucose concentrations. In addition, the response currents of the anodic and cathodic peaks increase with the increase of glucose concentrations. These results indicate good electrochemical sensing performance of porous NiMn_2_O_4_ NSs@NF electrode for glucose. For comparison, the electrochemical behaviors of porous NiMn_2_O_4_ NSs@NF electrode and bare nickel foam for the electrochemical sensing of glucose are investigated by CV technique at the sweep rate of 20 mV s^−1^ in a 0.5 M NaOH electrolyte solution. Figure 6d shows CV curves of porous NiMn_2_O_4_ NSs@NF electrode recorded in the presence of glucose and absence of glucose, and CV curve of bare nickel foam in the presence of glucose. The response current obtained on porous NiMn_2_O_4_ NSs@NF electrode with 1 mM glucose is 74.9 mA cm^−2^, which is much larger than that obtained on bare nickel foam electrode (4.6 mA cm^−2^). The result indicates the excellent electroactivity of porous NiMn_2_O_4_ NSs@NF electrode towards glucose. In the case of the presence of glucose, porous NiMn_2_O_4_ NSs@NF electrode also exhibits larger closed area than the bare nickel foam, indicating no obvious electrochemical response of bare nickel foam. In addition, porous NiMn_2_O_4_ NSs@NF electrode at the presence of glucose exhibits the high response current (74.9 mA cm^−2^) of the redox peaks compared to those of the absence of glucose (53.2 mA cm^−2^). This result also confirms the good electrochemical sensing performance of porous NiMn_2_O_4_ NSs@NF electrode for glucose. The high current response is attribute to the electroactivity by the large surface area, the outstanding electronic connectivity and the synergy effect of porous NiMn_2_O_4_ nanosheet arrays and nickel foam.

EIS is employed to test the electrochemical impedance property of porous NiMn_2_O_4_ NSs@NF sensor electrode. The electrochemical impedance test is employed in the frequency range from 10^5^ Hz to 10^−2^ Hz at 5 mV in a three-electrode cell with 0.5 M NaOH electrolyte solution. Figure S10 shows Nyquist plot of porous NiMn_2_O_4_ NSs@NF electrode. Nyquist plot shows a semicircle at higher frequencies and a long positive-slope line at the lower frequencies. This diameter of semicircle corresponds to the charge transfer resistance, indicating the electron transfer kinetics of the charge transfer process at the working electrode/electrolyte interface^65^. From the inset in Fig. S10, the small diameter of the semicircle reveals the good electric conductivity of porous NiMn_2_O_4_ NSs@NF electrode. At the lower frequencies, this positive-slope line corresponds to the Warburg impedance (Z_w_). From Nyquist plot, the slope line with an inclination angle approaching 60° reveals the good diffusion kinetics between the electrode surface and electrolyte^64,66^. The low electrochemical impedance indicates that porous NiMn_2_O_4_ NSs@NF electrode can provide an efficient electron transfer pathway and fast current response for glucose detection.

**Figure 7:**
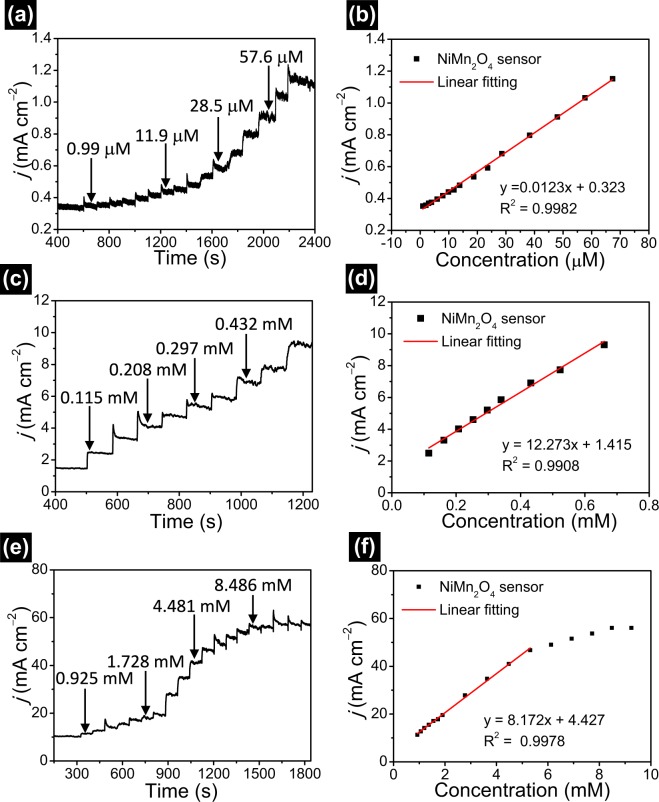
(**a**) The amperometric responses of porous NiMn_2_O_4_ NSs@NF sensor electrode with the successive additions of various concentration of glucose at a potential of 0.45 V (the concentration range of glucose in electrolyte bath is 0.99–67.30 μM). (**b**) The corresponding fitting curves, the concentration range of glucose in electrolyte bath is 0.99–67.30 μM, which shows the sensitivity 12.2 mA mM^−1^ cm^−2^. (**c**) The amperometric response with the successive additions of various concentration of glucose (the concentration range of glucose in electrolyte bath is 0.115–0.661 mM). (**d**) The corresponding fitting curves, the concentration range of glucose in electrolyte bath is 0.115–0.661 mM, which shows the sensitivity 12.3 mA mM^−1^ cm^−2^. (**e**) The amperometric response with the successive additions of various concentration of glucose (the concentration range of glucose in electrolyte bath is 0.925–8.486 mM). (**f**) The corresponding fitting curves, the concentration range of glucose in electrolyte bath is 0.067–1.373 mM.

The amperometric tests are performed to test the electrochemical response property of porous NiMn_2_O_4_ NSs@NF sensor electrode. Under optimal conditions, the amperometric responses are tested at a potential of +0.45 V in 0.5 M NaOH electrolyte solution. Figure 7 shows typical amperometric response curves for various concentrations of glucose. Figure 7a shows the amperometric responses of porous NiMn_2_O_4_ NSs@NF electrode with consecutive step changes of the glucose concentration at a potential of 0.45 V (the concentration range of glucose in electrolyte bath is 0.99–67.30 μM). As can be seen from Fig. 7a, porous NiMn_2_O_4_ NSs@NF electrode shows the good amperometric response at the glucose concentration range from 0.99 μM to 67.30 μM. Figure 7b shows the corresponding fitting curves of the amperometric responses vs. glucose concentrations (the concentration range is 0.99–67.30 μM). The amperometric response increases linearly with the increase of glucose concentration. The linear fitting regression equation is expressed as *y* (mA cm^−2^) = 0.01224*x* + 0.3228 (*R*^2^ = 0.9982). Porous NiMn_2_O_4_ NSs@NF electrode delivers a sensitivity of 12.2 mA mM^−1^ cm^−2^ at the window concentrations of 0.99–67.30 μM. In addition, the limit of detection (LOD) of glucose detection is calculated to be using the following equation^67^. LOD = 3 SD/S, where, S is the slope of the calibration curve (0.01224 mA μM^−1^ cm^−2^) and SD is the standard deviation of blank (9.9 × 10^−4^ mA cm^−2^). The detection limit is calculated to be 0.24 µM.

Figure 7c shows the amperometric response of porous NiMn_2_O_4_ NSs@NF electrode with consecutive step changes of the glucose concentration (the concentration range of glucose in electrolyte bath is 0.115–0.661 mM). Similarly, porous NiMn_2_O_4_ NSs@NF electrode shows the good amperometric response at the window concentrations of 0.115–0.661 mM. Figure 7d shows the corresponding fitting curves of the amperometric responses vs. glucose concentrations (the concentration range is 0.115–0.661 mM). The amperometric response increases linearly with the increase of glucose concentration with ranging from 0.115 mM to 0.661 mM. The linear fitting regression equation is expressed as *y* (mA cm^−2^) = 12.273*x* + 1.415 (*R*^2^ = 0.9908). Porous NiMn_2_O_4_ NSs@NF electrode delivers a sensitivity of 12.3 mA mM^−1^ cm^−2^ at glucose concentration ranging from 0.115 mM to 0.661 mM. Figure 7e shows the amperometric response of porous NiMn_2_O_4_ NSs@NF electrode with consecutive step changes of the glucose concentration (the concentration range is 0.925–8.486 mM). From Fig. 7e, porous NiMn_2_O_4_ NSs@NF electrode still exhibits the good amperometric response at concentration ranging from 0.925 mM to 8.486 mM. Figure 7f shows the corresponding fitting curves (the linear concentration range is 0.925–5.310 mM).

**Figure 8:**
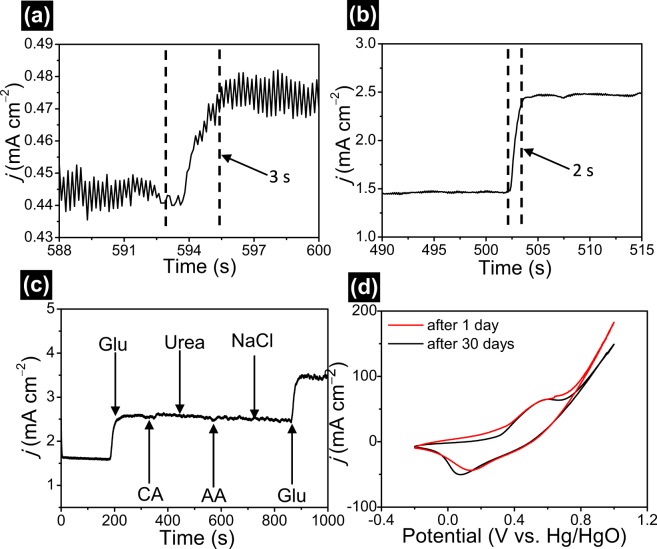
The response time of porous NiMn_2_O_4_ NSs@NF sensor electrode (**a**) A response time of 3 s at a gluconic concentration of 5.964 μM. (**b**) A response time of 2 s at a gluconic concentration of 0.115 mM. (**c**) Amperometric response of porous NiMn_2_O_4_ NSs@NF sensor electrode at 0.45 V upon additions of 1.0 mM glucose, 0.1 mM CA, 0.1 mM Urea, 0.1 mM AA, 0.1 mM NaCl and 0.1 mM Glu in 0.5 M NaOH electrolyte. (**d**) Comparing CV curves of porous NiMn_2_O_4_ NSs@NF sensor electrode after 1 day and 30 days at a sweep rate of 20 mV s^−1^ in 0.5 M NaOH with 1 mM glucose.

The amperometric response time is crucial parameter for the electrochemical sensor in non-enzymatic glucose detection. The response time of porous NiMn_2_O_4_ NSs@NF electrode is obtained by amperometric measurements in the different glucose concentration in 0.5 M NaOH electrolyte at 0.45 V. Figure 8 presents the response time of porous NiMn_2_O_4_ NSs@NF electrode. With the addition of glucose to electrolyte solution, the glucose oxidation current increases rapidly and then reaches to the steady state. The time begin from the current increase until the current signal to the stead state value is defined as the response time of the sensor. Figure 8a shows an observed response of the sensor is 3 s at a gluconic concentration of 5.964 μM. Figure 8b shows an observed response of the sensor is 2 s at a gluconic concentration of 0.115 mM, which is considered a quick response time. The quick amperometric response time is attributed to good sensibility, excellent electronic conductivity and efficient catalytic ability selectivity of porous NiMn_2_O_4_ NSs@NF electrode. The comparison for the sensing performances of porous NiMn_2_O_4_ NSs@NF electrode and other transition metal oxide materials is listed as shown in Table S1. This comparison table shows the excellent sensing performances of porous NiMn_2_O_4_ NSs@NF electrode compared to the reported sensor.

Anti-interference property of porous NiMn_2_O_4_ NSs@NF sensor electrode is crucial for non-enzymatic electrochemical detection of glucose. It is well-known that saccharides have similar electrochemical reaction behaviors or the interferential behaviors and chloride can lead to the catalyst poisoning to glucose detection. Thus, these compounds cannot be ignored. We investigate the amperometric responses from the other saccharides or chloride such as CA, urea, AA, NaCl in a 0.5 M NaOH electrolyte solution. With the addition of 1.0 mM glucose to the 0.5 M NaOH electrolyte, a distinct response current at 400 s can be observed from Fig. 8c. When upon addition of other interference compounds such as CA (0.1 mM), urea (0.1 mM), AA (0.1 mM), NaCl (0.1 mM), the current responses cannot be observed or the response current are acceptable and negligible compared to the response current of glucose molecules. With the additions of 1.0 mM glucose, two distinct amperometric responses at 200 s to 900 s can be observed toward glucose detection. The low current responses for other saccharides or chloride indicate that porous NiMn_2_O_4_ NSs@NF have the good selectivity for the electrochemical determination of glucose. Considering that the glucose level is at least 30~50 times higher than those of interfering species in human serum, these interference species produce negligible current responses compared with glucose molecules in a 0.5 M NaOH electrolyte solution^63^. Therefore, these result reveals that porous NiMn_2_O_4_ NSs@NF electrode will be well used toward the detection of glucose in practice.

The long-term stability of porous NiMn_2_O_4_ NSs@NF sensor electrode is examined after 30 days. The stability of porous NiMn_2_O_4_ NSs@NF electrode is measured by CV sweep at 20 mV s^−1^ in 0.5 M NaOH with 1 mM glucose. Figure 8d shows CV curves of porous NiMn_2_O_4_ NSs@NF electrode after 1 day and 30 days. From CV curves, we can observe that no distinct decrease for the peak current after 30-days storage. CV curves almost remains the same shape. In addition, the current response (64.5 mA cm^−2^) maintains 95.1% of the primitive response (67.8 mA cm^−2^) after one month storage. These results indicate the excellent electrochemical stability of porous NiMn_2_O_4_ NSs@NF sensor electrode.

## Conclusions

In summary, we have successfully fabricated porous NiMn_2_O_4_ NSs@NF sensor electrode *via* a facile hydrothermal reaction followed by a calcination. Porous NiMn_2_O_4_ NSs@NF electrode as non-enzymatic sensor for glucose detection was investigated by the characterizing the structure and electrochemical sensing performances. These porous NiMn_2_O_4_ nanosheets are directly grown on the surface of nickel foam substrate forming porous NiMn_2_O_4_ nanosheet arrays. Owing to the unique porous nanosheet arrays structure, porous NiMn_2_O_4_ NSs@NF electrode possesses large electrochemical active surface area, high electrochemical catalytic activity and fast electron-ion transfer process. The electrochemical tests show that porous NiMn_2_O_4_ NSs@NF as the non-enzymatic sensor electrode for glucose delivers good selective and stable, high sensitivity and reversible and fast response. The porous NiMn_2_O_4_ NSs@NF electrode exhibits the high sensitivity of 12.2 mA mM^−1^ cm^−2^ at a linear window concentrations ranging from 0.99 μM to 67.30 μM and 12.3 mA mM^−1^ cm^−2^ at a linear window concentrations ranging from 0.115 mM to 0.661 mM. The porous NiMn_2_O_4_ NSs@NF electrode also exhibits a low value of LOD (0.24 µM) and a fast response (2 s). These good electrochemical response performances for glucose indicate that porous NiMn_2_O_4_ NSs@NF electrode as non-enzymatic sensing material has the good potential and practical application prospects.

## Experimental Section

### Reagents

Nickel(II) nitrate hexahydrate (Ni(NO_3_)_2_·6H_2_O; ≥98.0%), potassium permanganate (KMnO_4_; ≥99.0%), urea (CO(NH_2_)_2_; ≥99.0%), glucose (C_6_H_12_O_6_·H_2_O; α_D_: +52.5~+53.0°), ascorbic acid (C_6_H_8_O_6_, AA; ≥99.7%) and citric acid (C_6_H_8_O_7_, CA; ≥99.5%) were purchased and obtained from Tianjin Guangfu Technology Development Co. Ltd.. Hydrochloric acid (HCl, 36.0~38.0%) was obtained and purchased from Jinzhou Ancient City Chemical Reagents Factory. Sodium chloride (NaCl; ≥99.5%) and sodium hydroxide (NaOH; ≥96.0%) were obtained and purchased from Tianli Chemical Reagent Co. Ltd.. Ammonium fluoride (NH_4_F; ≥98.0%) was obtained and purchased from Tianjin Fuchen Chemical Reagents Factory. Nickel foam was purchased and obtained from Taiyuan Liyuan Lithium Technology Co. Ltd., more detailed technical parameters of nickel foam were shown in Table S2. De-ionized water (18.3 MΩ cm at 25 °C) was purified and obtained by Milli-Q water system to prepare all solutions. In our work, all chemical reagents and materials were also used without further purification unless otherwise described.

### Fabrication of porous NiMn_2_O_4_ NSs@NF electrode

The typical procedure, porous NiMn_2_O_4_ NSs@NF were fabricated via a simple hydrothermal method followed by a heat treatment. Briefly, 2 mmol Ni(NO_3_)_2_·6H_2_O (0.582 g), 12 mmol NH_4_F (0.444 g) and 30 mmol CO(NH_2_)_2_ (1.800 g) were dissolved into 40 mL deionized water under continuous electromagnetic stirring. A nickel foam (length × width × thickness = 20 mm × 10 mm × 1 mm) was treated and purified with 3 M HCl for 15 min for the remove of the oxide layer on nickel foam. Then, the acid-treated nickel foam was cleaned sequentially by copious amounts of de-ionized water. Then, this pre-treated nickel foam was immersed into above mixed solution. 4 mmol KMnO_4_ (0.632 g) was dissolved into 40 mL de-ionized water and then was added into the previous solution. After stirring for 30 min, the mixed solution was transferred into a 100 ml-Teflon-lined stainless steel hydrothermal reactor and followed by heating at 110 °C for 8 h. After cooling to the ambient temperature, the precursor was collected and washed with de-ionized water and ethanol thoroughly to remove residual ions. The precursor was dried at 60 °C for 2 h. Finally, the precursor was converted to porous NiMn_2_O_4_ NSs@NF under 350 °C for 2 h and then naturally cooled to the ambient temperature.

### Instruments and characterizations

X-ray diffraction (XRD) measurement was carried out using the Rigaku RAD-3C diffractometer instrument (Cu Kα, *λ* = 1.5405 Å, 35 kV, 20 mA, *2-Theta* angles: 10°–70°). The morphology and structure were investigated by scanning electron microscopy (SEM, JEOL S-4800) under the condition of 3.0 kV operating voltage. Transmission electron microscopy (TEM, JEOL JEM-2100F microscopy) with an energy dispersive X-ray spectroscope (EDS) was also carried out to investigate the element distributions of porous NiMn_2_O_4_ NSs@NF under the condition of 200 kV accelerating voltage. X-ray photoelectron spectroscopy (XPS, ESCALB-MKII250) was performed to analyze the elemental compositions and its valence of porous NiMn_2_O_4_ NSs@NF under a monochromatic 150 W Al Kα source radiation. Nitrogen adsorption/desorption measurement was performed to analyze the specific surface area of porous NiMn_2_O_4_ NSs@NF on a Micromeritics ASAP 2010 analyzer at 77 K.

### Electrochemical measurements

All electrochemical measurements of porous NiMn_2_O_4_ NSs@NF were tested and executed in a three−electrode system cell by an electrochemical workstation (CHI660D, Shanghai, China). In the detecting process, porous NiMn_2_O_4_ NSs@NF was directly used as a sensor electrode. The Hg/HgO electrode with 1 M KOH electrolyte was used as the reference electrode. Platinum plate (length × width × thickness = 20 mm × 20 mm × 0.2 mm) was used as the counter electrode. The 0.5 M NaOH aqueous with different concentration glucose solutions were used as the electrolyte in the electrochemical test process. Cyclic voltammetry (CV), single-potential amperometry and electrochemical impedance spectroscopy (EIS) technologies were carried out to investigate the electrochemical response performances of porous NiMn_2_O_4_ NSs@NF electrode. CV and amperometry measurements were carried out under the magnetic stirring condition of 400 rpm. EIS test was carried out without the stirring condition. All electrochemical measurements were executed at the ambient temperature of 25 °C.

## Supplementary information

Supplementary Information
