# Supplementary material for: Fabrication of porous NiMn2O4 nanosheet arrays on nickel foam as an advanced sensor material for non-enzymatic glucose detection
Source: Sci Rep. 2019 Dec 2;9:18121. doi: 10.1038/s41598-019-54746-2 (PMC6889510; doi:10.1038/s41598-019-54746-2)
Supplement: Supplementary file 1 — Supplementary Information [file 41598_2019_54746_MOESM1_ESM.docx]

**Supplementary Information**

**Fabrication of porous NiMn_2_O_4_ nanosheet arrays on nickel foam as an advanced sensor material for non-enzymatic glucose detection**

Jie Zhang*^1^*, Yudong Sun*^2^*, Xianchun Li*^1^**, Jiasheng Xu*^2^***

*^1^School of Chemical Engineering, University of Science and Technology Liaoning, Anshan 114051, P.R. China.*

*^2^Liaoning Province Key Laboratory for Synthesis and Application of Functional Compounds, College of Chemistry and Chemical Engineering, Bohai University, Jinzhou 121013, P.R. China.*

*Correspondence and requests for materials should be addressed to X. L. (email:* *xianchunli@ustl.edu.cn) or J. X. (email:* [*jiashengxu@bhu.edu.cn*](mailto:liuxy@jlu.edu.cn)*)*

**
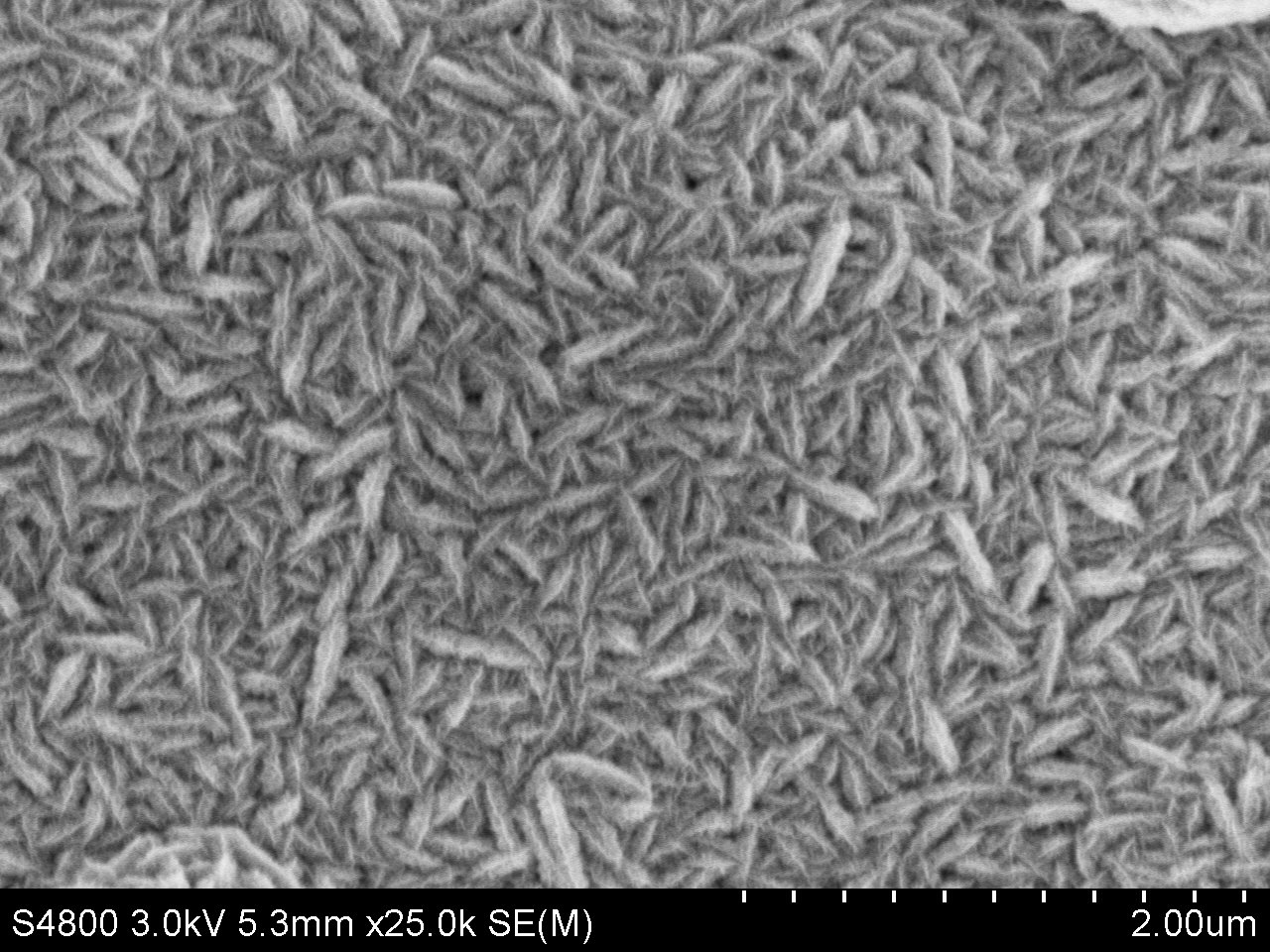
**

**Figure S1.** SEM image of the porous NiMn_2_O_4_ nanosheet arrays.

**
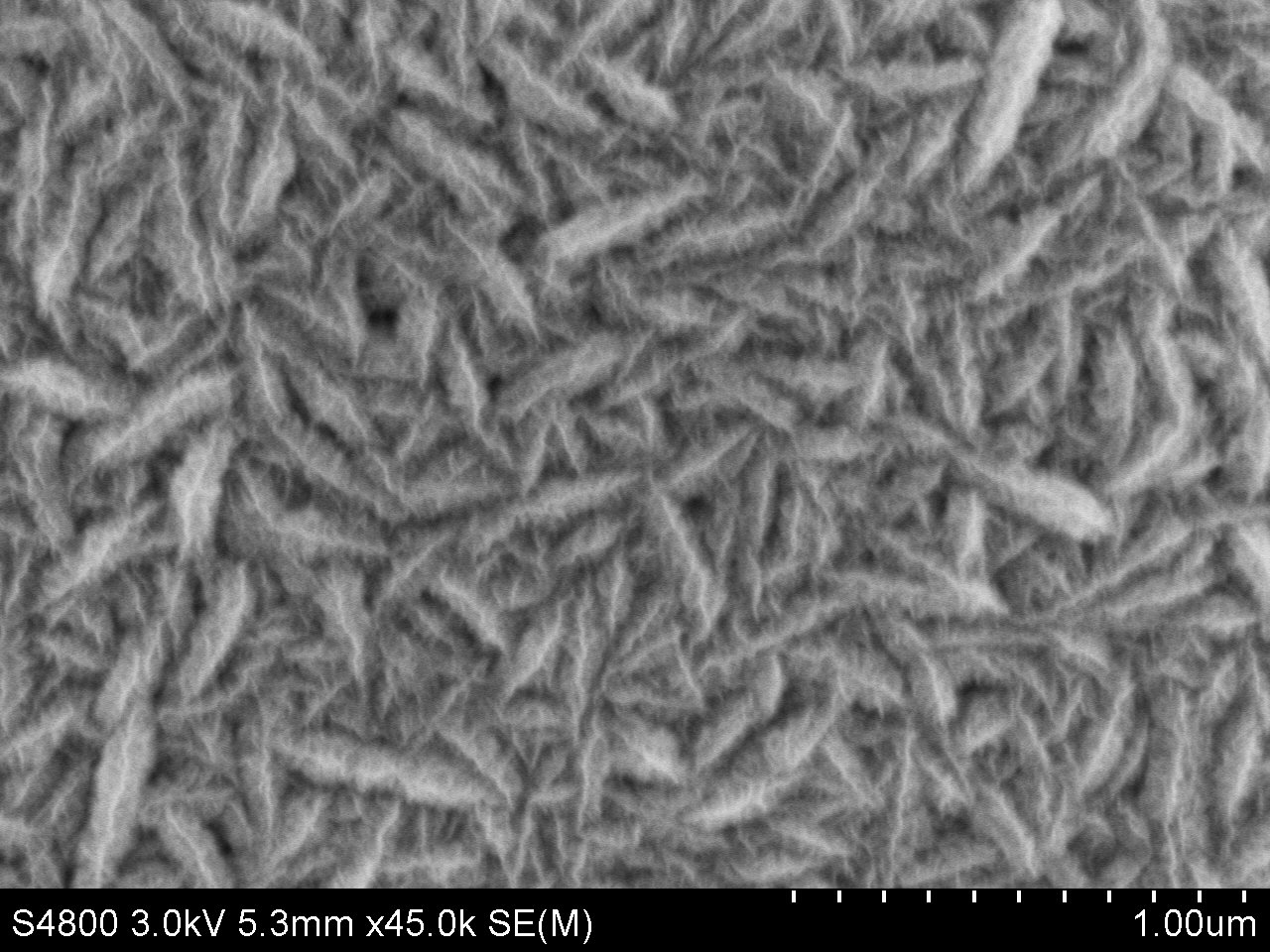
**

**Figure S2.** SEM image of the porous NiMn_2_O_4_ nanosheet arrays.

**
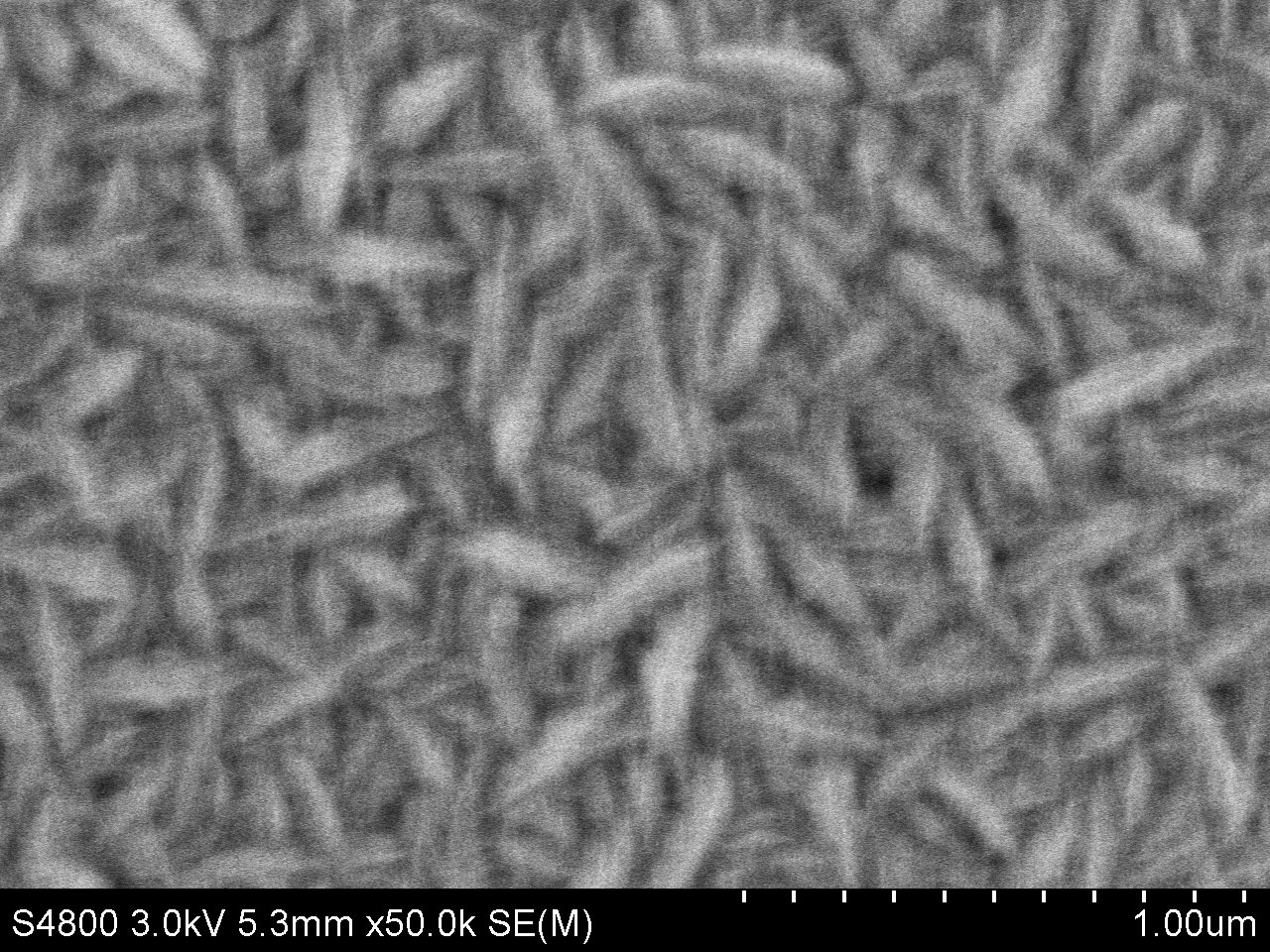
**

**Figure S3.** SEM image of the porous NiMn_2_O_4_ nanosheet arrays.

**
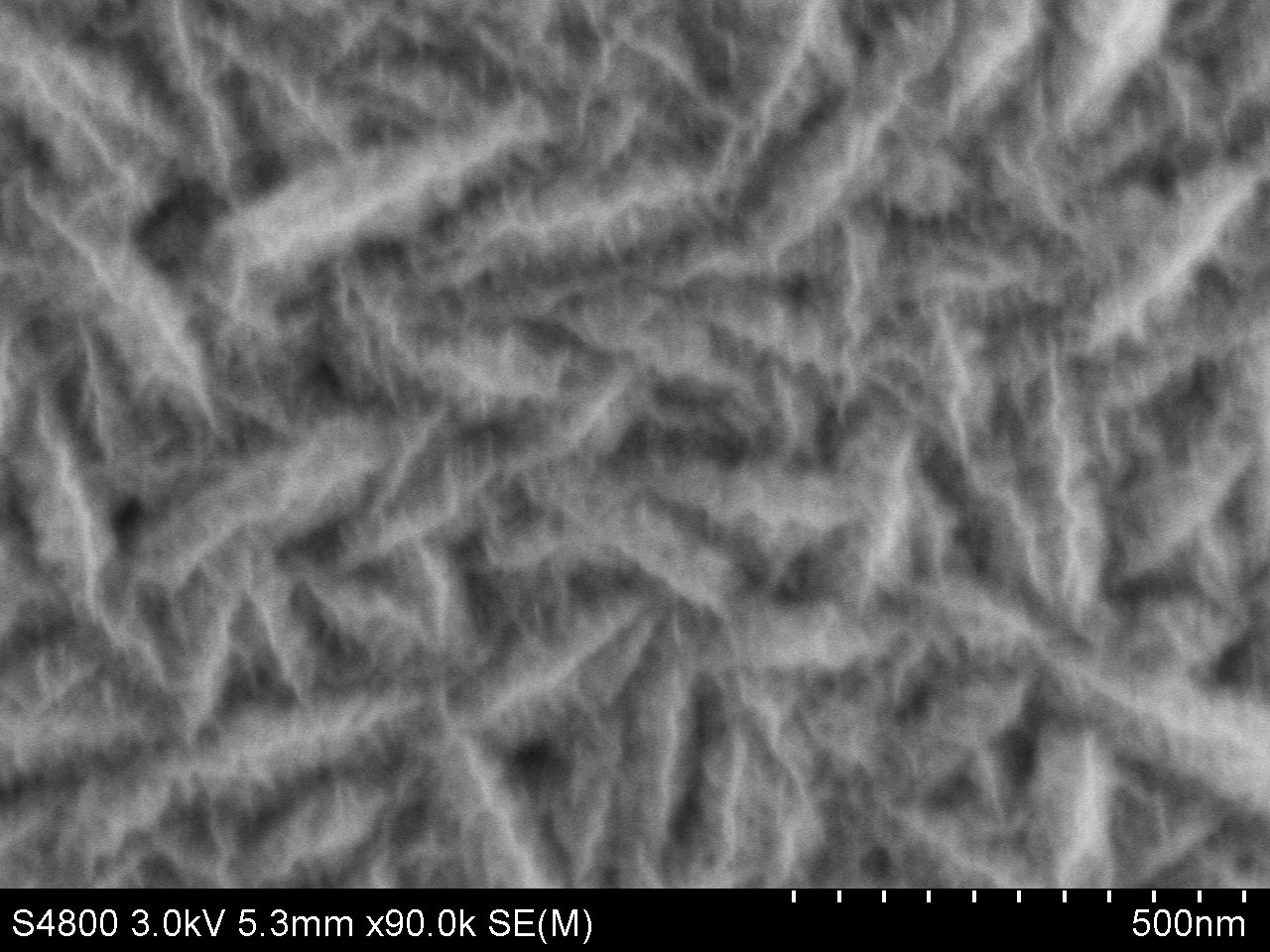
**

**Figure S4.** SEM image of the porous NiMn_2_O_4_ nanosheet arrays.

**
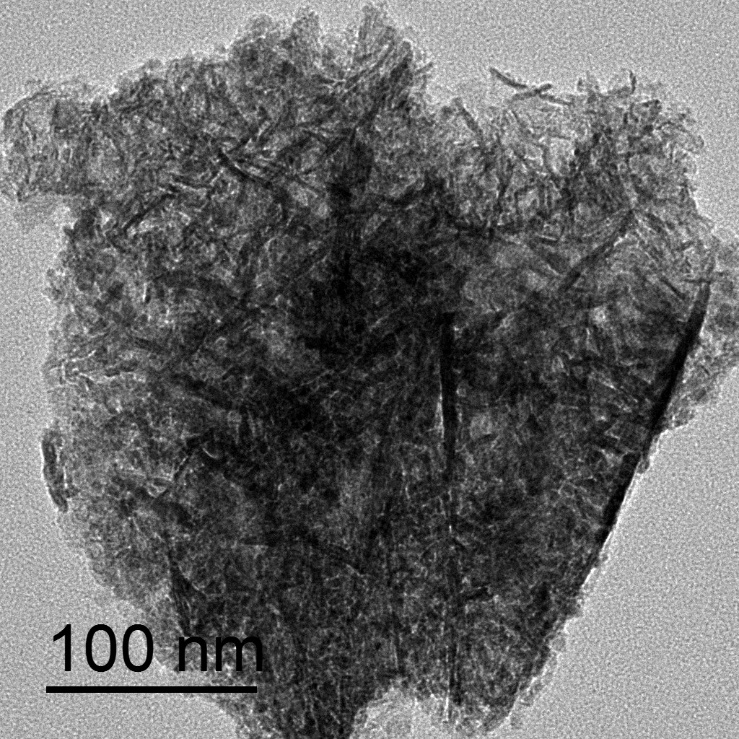
**

**Figure S5.** TEM image of the porous NiMn_2_O_4_ nanosheet, which shows the integrated NiMn_2_O_4_ nanosheet.

**
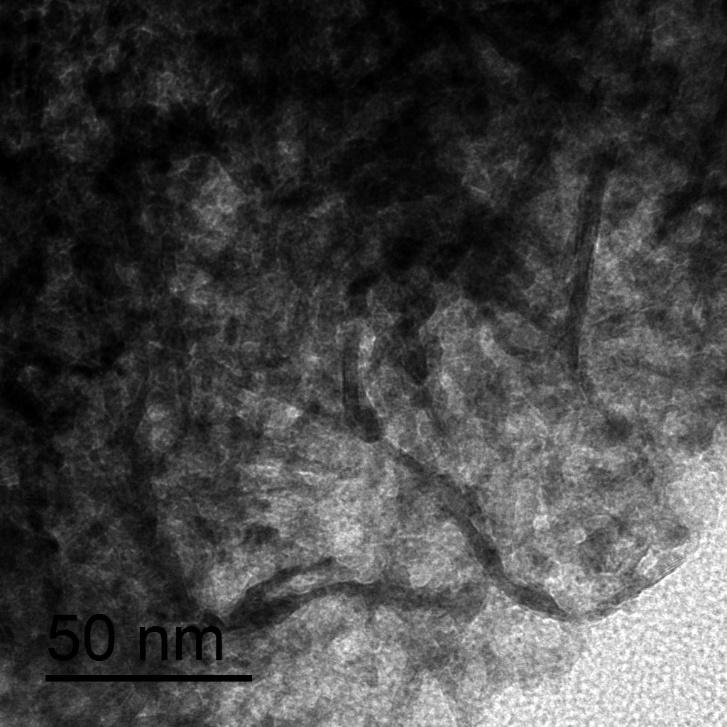
**

**Figure S6.** TEM image of the porous NiMn_2_O_4_ nanosheet

**
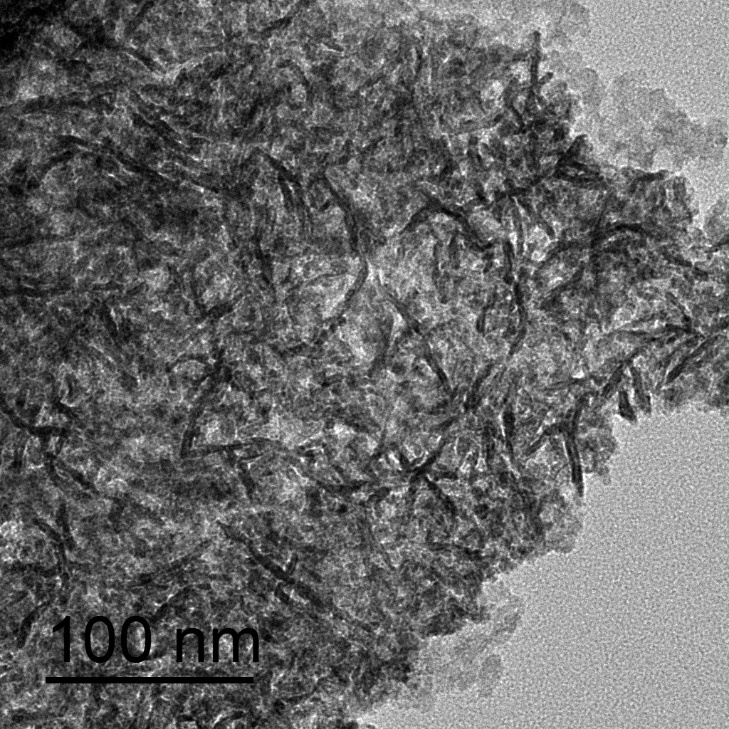
**

**Figure S7.** TEM image of the porous NiMn_2_O_4_ nanosheet


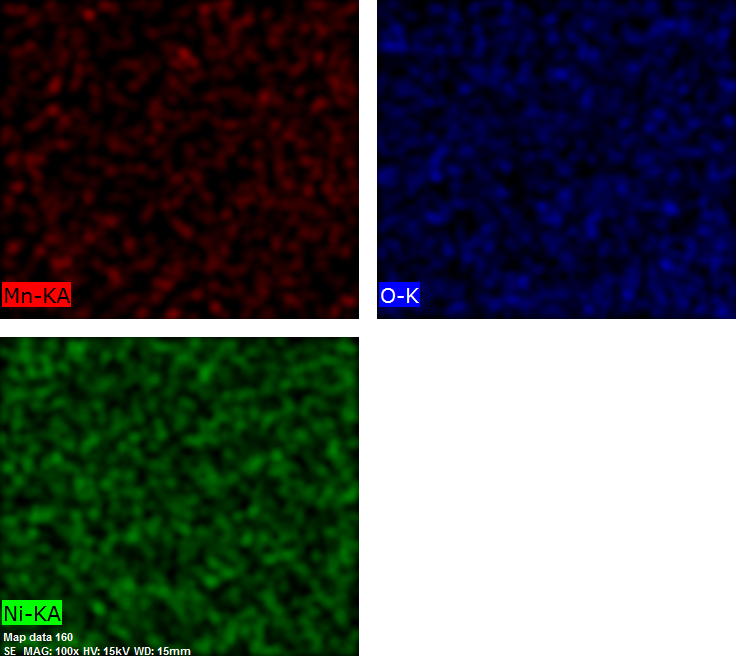

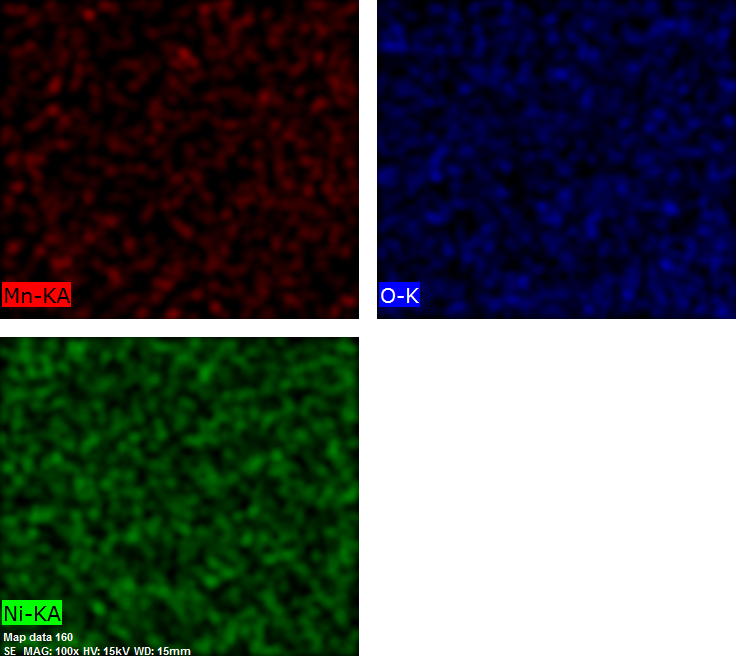

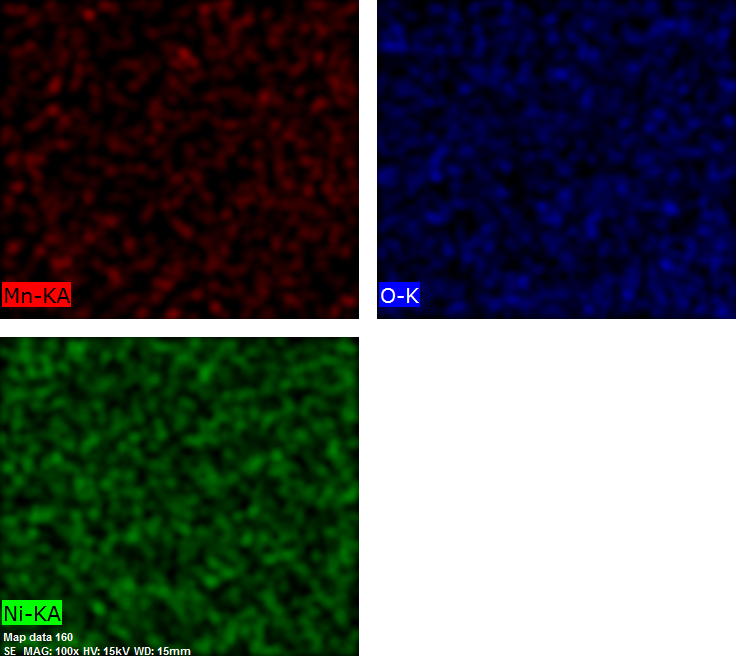

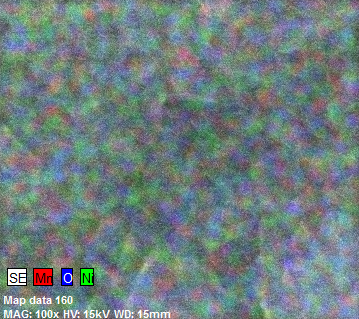


**(a)**

**(b)**

**(c)**

**(d)**

**Figure S8.** The energy dispersive spectroscopy (EDS) mapping of porous NiMn_2_O_4_ NSs@NF sensor electrode under the condition of 20.0 kV operating voltage. (a) Overlapped elemental mapping image of Ni, Mn and O. (b) Ni mapping. (c) Mn mapping. (d) O mapping.

9.6 nm

S_BET_=77.5 m^2^ g^−1^

**Figure S9.** Brunauer-Emmett-Teller (BET) nitrogen adsorption and desorption isotherms of porous NiMn_2_O_4_ NSs@NF. Inset is the pore size distribution plot with based on BJH measurement. BET measurement is conducted in liquid nitrogen bath with the mixed of nitrogen (99.999%) and helium at the temperature of 77.4 K.

**Figure S10.** Nyquist plots of porous NiMn_2_O_4_ NSs@NF sensor electrode in the frequency range from 0.01 Hz to 100 kHz under an amplitude of 0.005 A.

**Table S1.** Comparison of the electrochemical performance of glucose detection for the porous NiMn_2_O_4_ nanosheet arrays on nickel foam sensor in this work and the previously reported transition metal based sensors.

| Electrode  materials | Sensitivity  mA mM^−1^ cm^−2^ | Linger range  μM | | LOD  μM | | Response time | | reference |
| --- | --- | --- | --- | --- | --- | --- | --- | --- |
| NiCo_2_O_4_ nanosheet on ITO ^a^ | 6.69 | | 6–65 | | 0.38 | | 26 | [^1^](#_ENREF_1) |
| NiCo_2_O_4_ nanorods | 4.71 | | 1–880 | | 0.063 | | 3 | [^2^](#_ENREF_2) |
| NiO nanosheets/CPE ^b^ | 0.74 | | 50–3000 | | 10 | | - | [^3^](#_ENREF_3) |
| NiCo_2_O_4_@Polyaniline | 4.55 | | 15–4735 | | 0.3833 | | 5 | [^4^](#_ENREF_4) |
| Porous NiCo_2_O_4_ nanospheres | 1.917 | | 10–2240 | | 0.6 | | 10 | [^5^](#_ENREF_5) |
| Co_3_O_4_/NiCo_2_O_4_ nanocages | 0.304 | | 10–3520 | | 0.384 | | 4 | [^6^](#_ENREF_6) |
| rGO-NiCo_2_O_4_ nanorods ^c^ | 0.960 | | 1–25000 | | 0.35 | | 5 | [^7^](#_ENREF_7) |
| NiCo_2_O_4_ hollow nanorods | 1.685 | | 0.3–1000 | | 0.16 | | - | [^8^](#_ENREF_8) |
| MoS_2_-NiCo_2_O_4_ | 1.748 | | 1-1600 | | 0.152 | | - | [^9^](#_ENREF_9) |
| NiMn_2_O_4_/rGOHs | 1.310 | | 2–20000 | | 1.78 | | 3.5 | [^10^](#_ENREF_10) |
| NiO/SiC | 2.037 | | 4−7500 | | 0.32 | | - | [^11^](#_ENREF_11) |
| IrO_2_@NiO/GCEs ^d^ | 1.439 | | 5–2500 | | 0.31 | | - | [^12^](#_ENREF_12) |
| α-MnO_2_/Co_3_O_4_ | 0.127 | | 60–7000 | | 0.03 | | 5 | [^13^](#_ENREF_13) |
| Ni(OH)_2_ on Ni microflowers | 2.392 | | 0.5–2995 | | 0.0024 | | - | [^14^](#_ENREF_14) |
| Coral-like PtAu–MnO_2_ | 0.058 | | 100–4000 | | 20 | | - | [^15^](#_ENREF_15) |
| MnO_2_ micro/nanorod arrays | 1.650 | | 10–3000 | | 1.9 | | 3 | [^16^](#_ENREF_16) |
| Porous NiMn_2_O_4_ NS@NF | 12.2  12.3 | | 0.99–67.3  115–661 | | 0.24 | | 2 | This work |

^a^ Indium tin oxide; ^b^ carbon paste electrode; ^c^ reduced graphene oxide; ^d^ reduced graphene oxide hydrogel.

**Table S2.** [Nickel foam](https://www.amazon.com/capacitor-specific-battery-grade-capacitor-electrode-0-5x250x200mm/dp/B06VTZ7S6X/ref=sr_1_1?ie=UTF8&qid=1513582119&sr=8-1&keywords=Ni+foam#productDetails) technical parameters

| Technical parameters |  |
| --- | --- |
| Ni purity  Porosity  Strength of extension  Bore diameter  PPI  Thickness  Flexibility  Appearance  The surface density | 99.8%  95%, up to 98%  >1 Mpa  0.2-0.6 mm  110  1.0 mm  round volume winding a circle, not broken φ6 mm  Silver grey metallic luster  300 g/m^2^ ± 20 g/m^2^ |

**References**

1. Naik, K.K. et al. Electrodeposited spinel NiCo_2_O_4_ nanosheet arrays for glucose sensing application. *RSC Adv.* **5**, 74585-74591 (2015).

2. Saraf, M. et al. Multifunctional porous NiCo_2_O_4_ nanorods: sensitive enzymeless glucose detection and supercapacitor properties with impedance spectroscopic investigations. *New J. Chem.* **41**, 9299-9313 (2017).

3. Ibrahim, A.A. et al. Highly sensitive and selective non-enzymatic monosaccharide and disaccharide sugar sensing based on carbon paste electrodes modified with perforated NiO nanosheets. *New J. Chem.* **42**, 964-973 (2018).

4. Yu, Z. et al. Facile synthesis of NiCo_2_O_4_@Polyaniline core-shell nanocomposite for sensitive determination of glucose. *Biosens. Bioelectron.* **75**, 161-165 (2016).

5. Huang, W. et al. Fast synthesis of porous NiCo_2_O_4_ hollow nanospheres for a high-sensitivity non-enzymatic glucose sensor. *Appl. Surf. Sci.* **396**, 804-811 (2017).

6. Xue, B. et al. Graphene wrapped porous Co_3_O_4_/NiCo_2_O_4_ double-shelled nanocages with enhanced electrocatalytic performance for glucose sensor. *Electrochim. Acta* **239**, 36-44 (2017).

7. Ni, Y. et al. Fabrication of RGO-NiCo_2_O_4_ nanorods composite from deep eutectic solvents for nonenzymatic amperometric sensing of glucose. *Talanta* **185**, 335-343 (2018).

8. Yang, J. et al. Synthesis of hierarchical NiCo_2_O_4_ hollow nanorods via sacrificial-template accelerate hydrolysis for electrochemical glucose oxidation. *Biosens. Bioelectron.* **75**, 15-22 (2016).

9. Wang, S. et al. MoS_2_ as connector inspired high electrocatalytic performance of NiCo_2_O_4_ nanoplates towards glucose. *Sens. Actuators B Chem.* **254**, 1101-1109 (2018).

10. Yen-Linh Thi Ngo et al. NiMn_2_O_4_ spinel binary nanostructure decorated on three-dimensional reduced graphene oxide hydrogel for bifunctional materials in non-enzymatic glucose sensor. *Nanoscale* **9**, 19318-19327 (2017).

11. Yang, P. et al. NiO/SiC nanocomposite prepared by atomic layer deposition used as a novel electrocatalyst for nonenzymatic glucose sensing. *ACS Appl. Mater. Interfaces* **7**, 4772-4777 (2015).

12. Wang, J. et al. Engineered IrO_2_@NiO core–shell nanowires for sensitive non-enzymatic detection of trace glucose in saliva. *Anal. Chem.* **88**, 12346-12353 (2016).

13. Sinha, L. et al. Hybridization of Co_3_O_4_ and α-MnO_2_ nanostructures for high-performance nonenzymatic glucose sensing. *ACS Sustain. Chem. Eng.* **6**, 13248-13261 (2018).

14. Manikandan, A. et al. Low-temperature chemical synthesis of three-dimensional hierarchical Ni(OH)_2_-coated Ni microflowers for high-performance enzyme-free glucose sensor. *J. Phys. Chem. C* **120**, 25752-25759 (2016).

15. Xiao, F. et al. Growth of coral-like PtAu-MnO_2_ binary nanocomposites on free-standing graphene paper for flexible nonenzymatic glucose sensors. *Biosens. Bioelectron.* **41**, 417-423 (2013).

16. Weina, X. et al. A novel β-MnO_2_ micro/nanorod arrays directly grown on flexible carbon fiber fabric for high-performance enzymeless glucose sensing. *Electrochim. Acta* **225**, 121-128 (2017).
